# Supplementary material for: Assessing Automated Facial Action Unit Detection Systems for Analyzing Cross-Domain Facial Expression Databases
Source: Sensors (Basel). 2021 Jun 20;21(12):4222. doi: 10.3390/s21124222 (PMC8235167; doi:10.3390/s21124222)
Supplement: Supplementary file 1 [file sensors-21-04222-s001.zip › sensors-1218699-supplementary.pdf]

Characteristics of Target Stimuli

All frequencies of manually coded AUs are described in Figure S1. As shown, the AU frequencies of wild data (Aff-wild2) were low except for AU1 (inner brow raiser). The muscles around the mouth, eyes, and cheeks (e.g., cheek raiser, AU6; lid tightener, AU7, lip corner puller, AU12; chin raiser, AU17) frequently occurred during conversation (GFT), and the AUs in posed expressions (DISFA+) were expressed in a well-balanced manner.

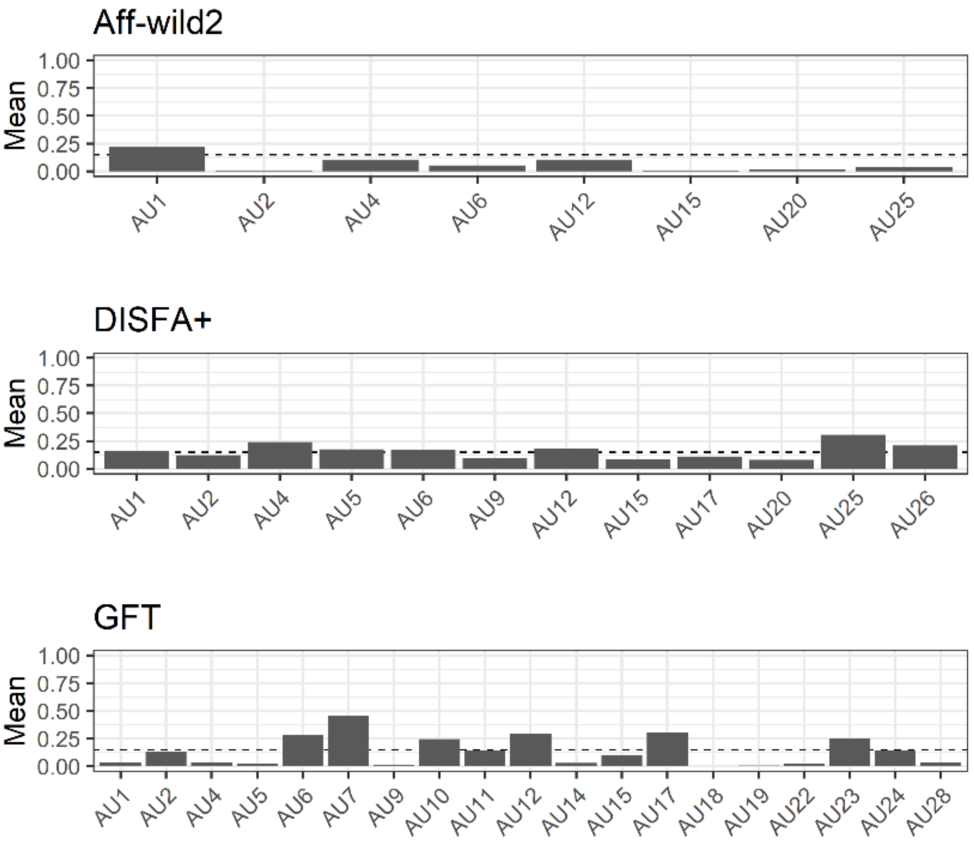

Figure S1. The frequencies of each manually coded AU in all databases. The dotted line represents 20%.

Additionally, the correlation matrix showing the co-occurrence of AUs in all three databases is provided in Figure S2. AU6 (cheek raiser), 7 (lid tightener), 10 (upper lip raiser), and 12 (lip corner puller) seem to co-occur. AU17 (chin raiser) and 24 (lip pressor), AU25 (lips part) and 26 (jaw drop) are also likely to co-occur.

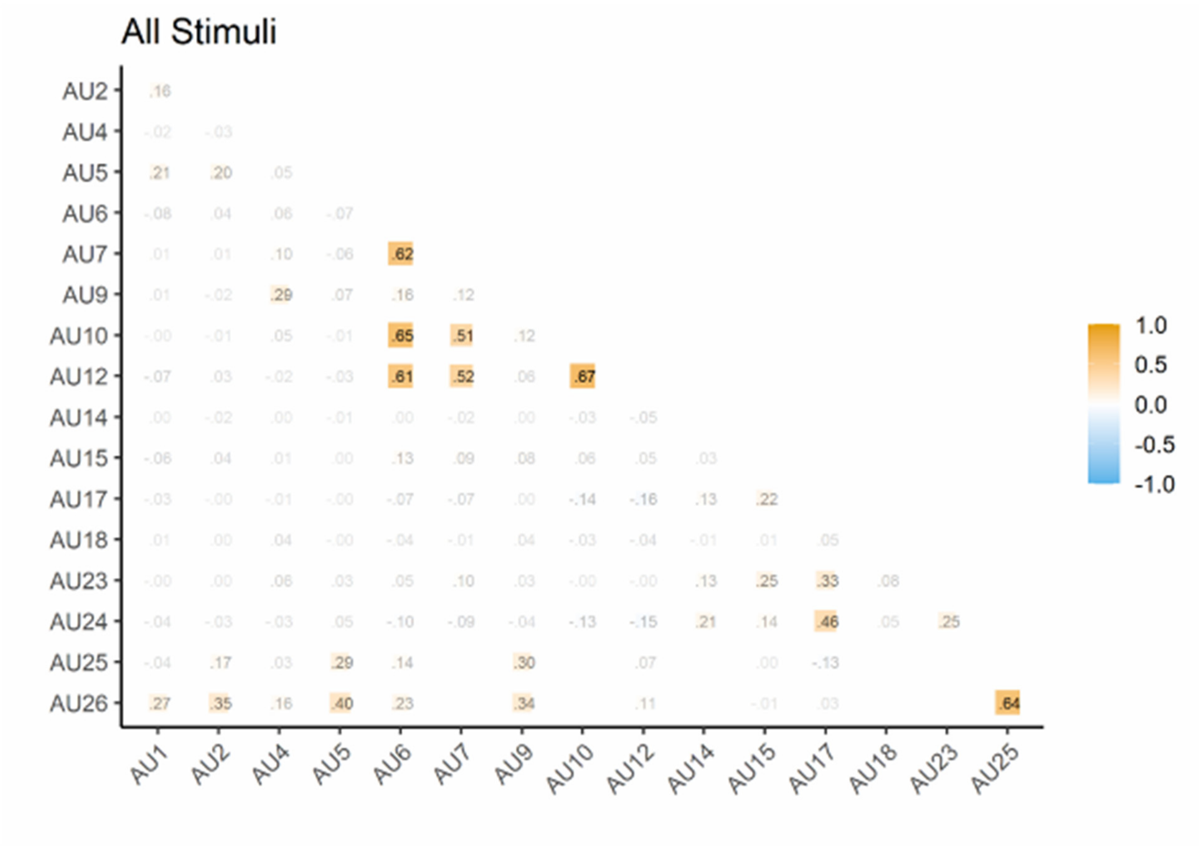

Figure S2. The correlation matrix for AUs in all three databases. If this value is close to 1, two AUs co-occurred. Orange squares represent positive coefficients, and blue squares represent negative ones.

Evaluation metrics

Using R statistical software, version 4.0.3 (https ://www.r-proje ct.org/) alongside the "caret" and "tidyverse" packages (Kuhn, 2020; Wickham et al., 2019), we calculated a confusion matrix, resulting in related statistics: sensitivity, specificity, prevalence, PPV, NPV, detection rate, detection prevalence, balanced accuracy, precision, recall, and F1 score. For details of the formulas, please refer to https://rdrr.io/cran/caret/man/confusionMatrix.html. In addition, negative agreement (NA), which evaluates the solution by the harmonic agreement of samples not including AUs (Ertugrul et al., 2020), was included.

All evaluation metrics are described at https://osf.io/fqh4g/?show=view

Expression Comparisons

To provide further information for affective computing, we compared the AUC values obtained by tools after grouping videos by emotions in the DISFA+ posed facial database. The raw data are available at OSF (https://osf.io/7rguf/?show=view). In two-way ANOVA, the factors were the tools (AFAR, FaceReader and OpenFace) and emotions (anger, disgust, fear, happiness, sadness and surprise). There was no main effect of tool, $F(3, 168) = 182.12$, $p < 0.001$, $\eta G2 = 0.68$ or emotion, $F(5, 168) = 3.08$, $p = 0.01$, $\eta G2 = 0.08$, and no significant tool × emotion interaction effect, $F(10, 168) = 1.51$, $p = 0.14$, $\eta G2 = 0.08$.

The main effect of the tool was consistent with the main analysis in the original paper. Shaffer's modified sequentially rejective Bonferroni procedure revealed that the AUC values for OpenFace (Mean = 0.81) and AFAR were higher than for FaceReader, *t*s > 13.49, *p*s < 0.001, *g*s > 2.60, and that the AUC value for anger was higher than that for happiness, $t(61) = 3.36$, $p = .01$, *Hedge's g* [95% CI] = 0.84 [0.33, 1.36].

References

1. Ertugrul, I.O.; Cohn, J.F.; Jeni, L.A.; Zhang, Z.; Yin, L.; Ji, Q. Crossing domains for AU coding: Perspectives, approaches, and measures. *IEEE Trans. biometrics Behave. Identity Sci.* **2020,** *2*, 158–171. doi: 10.1109/TBIOM.2020.2977225
2. Caret: Classification and Regression Training. R package version 6.0–86. Available online: https://CRAN.R-project.org/package=caret
3. Wickham, H.; Averick, M.; Bryan, J.; Chang, W.; McGowan, L.D.A.; François, R.; Grolemund, G.; Hayes, A.; Henry, L.; Hester, J.; Kuhn, M. Welcome to the Tidyverse. *J.Open Source Software* **2019,** *44*, 1686. doi: /10.21105/joss.01686
